# Sustainable Flame-Retardant Additives for Polymers: Future Perspectives

**DOI:** 10.3390/polym15061469

**Published:** 2023-03-16

**Authors:** Mohammad Reza Saeb, Henri Vahabi

**Affiliations:** 1 Department of Polymer Technology, Faculty of Chemistry, Gdańsk University of Technology, Gabriela Narutowicza 11/12, 80-233 Gdańsk, Poland; 2 Advanced Materials Center, Gdańsk University of Technology, Gdańsk, Poland; 3 LMOPS, Université de Lorraine, CentraleSupélec, F-57000 Metz, France

The increased use of plastics, particularly in terms of the use of polymers in electronics and electrical devices commonly used in homes, offices, schools, restaurants, and vehicles, has caused increased fire risks. In this sense, fire safety regulations are similarly becoming increasingly prevalent and relied on for the protection of buildings and human beings alike against fire through the intelligent management and organization of flame-retardant polymer materials. Several classes of flame-retardant (FR) additives have been under progressive development over the last few decades to help save lives. The selection of FR additives has represented another serious challenge faced by materials scientists and technologists because of the difficulties associated with the low thermal stability and processability of polymers comprising FR additives; the high costs of efficient FR additives; and, more critically, the drawbacks related to health and environmental toxicity [1]. Local legislations and rules, along with social and political issues, might cause additional complexities for, and criticisms of, manufacturers. Among the main concerns shared by all nations about fire risks is the toxicity of some halogenated FR additives and their harmful impacts to the environment, along with the limited biodegradability and sporadic recycling of flame-retardant polymers. Thus, attention has been directed towards the development of sustainable FR additives for polymers. 

Science, industry, society, and policy are the four pillars or main actors of the sustainable development of FR additives. The harmonization of sustainability concerns is dependent on the realization and recombination of the interface between economic growth, social welfare, and environmental protection—in which both theoretical and practical concepts are required [2]. Some bio-based FR additives have been more recently used instead of systems that are non-degradable or have limited degradability, in line with the global focus on finding replacements for fossil-based materials [3]. Typically, the vast majority of bio-based FR additives originate from biomass or animals [4]. From a practical viewpoint, green chemistry can derive the usage as well as the synthesis of bio-based FR additives, but the extent to which it appears useful and efficient is pertinent to the execution and implementation of sustainable regulations in charge of ecological design (ecodesign), which are altogether fueled by sustainable conceptualization (Figure 1). Not surprisingly, in some regions such as North America, the aforementioned sectors of fire safety realm continue to struggle with being exempted under existing instructions, which means that sustainability concerns are not considered in decision-making strategies. Thus, there is lack of trade-off between the environmental, human health, and engineering requirements in the design and commercialization of sustainable FR additives. 

Indeed, the future of FR additives should be supported by the principles of ecodesign. Moreover, an amalgamation of policies should preferably result in the regular cooperation of countries, and more globally, all continents should increase the radius of agreement in the quest for the integration of regulations and ideas into sustainable protocols for designing and manufacturing greener and cleaner FR additives for polymers. 

## Figures and Tables

**Figure 1:**
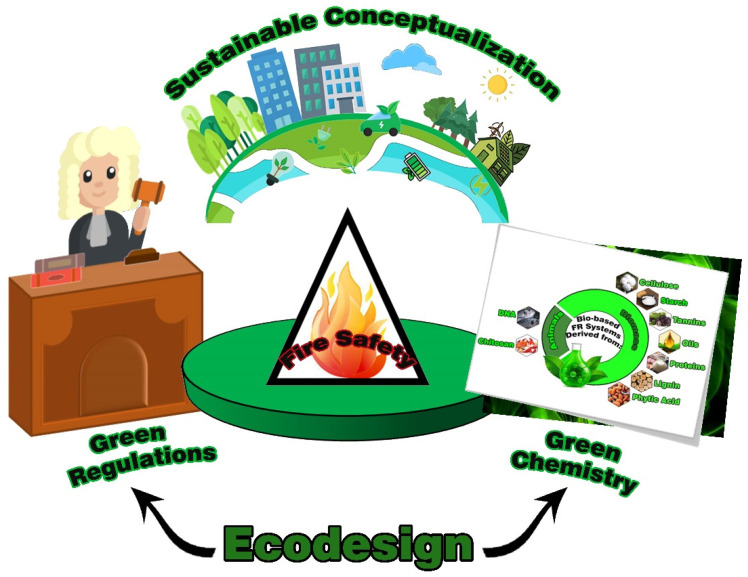
Triangular overview of development of sustainable FR additives for polymers. Science, industry, society, and policy are actors of sustainable development of FR additives. As roughly shown here, a thinking system conceptualizes sustainable ideas to bridge the ways green chemistry (science) and green regulation (policy) disciplines can be integrated into a practical guideline highlighting the role and implementing the concept of ecodesign (industry and society).
